# Bacteria Producing Extended Spectrum β-lactamases (ESBLs) in Hospitalized Patients: Prevalence, Antimicrobial Resistance Pattern and its Main Determinants

**DOI:** 10.30699/IJP.14.1.61

**Published:** 2018-12-27

**Authors:** Mehdi Yousefipour, Mehrnaz Rasoulinejad, Azar Hadadi, Negin Esmailpour, Alireza Abdollahi, Sirous Jafari, Atieh Khorsand

**Affiliations:** 1 *Dept. of Infectious and Tropical Diseases, Imam Khomeini Hospital Complex, Tehran University of Medical Sciences, Tehran, Iran*; 2 *Professor, Dept. of Infectious Diseases, Imam Khomeini Complex Hospital, Tehran University of Medical Sciences, Tehran, Iran*; 3 *Professor, Sina Hospital, Tehran University of Medical Sciences, Tehran, Iran*; 4 *Associate Professor, Dept. of Infectious Diseases, Shariati Hospital, Tehran University of Medical Sciences, Tehran, Iran*; 5 *Professor, Dept. of Pathology, Imam Khomeini Hospital Complex, School of Medicine, Tehran University of Medical Sciences, Tehran, Iran*; 6 *Associate Professor, Dept. of Infectious Diseases, Imam Khomeini Complex Hospital, Tehran University of Medical Sciences, Tehran, Iran*; 7 *Dept. of Pathology, Shariati Hospital Complex, School of Medicine, Tehran University of Medical Sciences, Tehran, Iran*

**Keywords:** beta-Lactamase, Antibiotic Resistance Hospital-Patient

## Abstract

**Background and Objective:**

There is a growing concern regarding the lack of new antibiotics, especially for multidrug- resistant bacteria that produce Extended Spectrum *β*-Lactamases (ESBLs). The present study aims to assess the preva- lence of bacteria producing ESBLs, their antimicrobial resistance pattern, and its main determinants among hospitalized patients.

**Methods:**

The study population included 383 consecutive patients with a definitive diagnosis of urinary tract infection (UTI). All eligible subjects for the study had a positive culture for gram-negative microorganisms in urine specimens. ESBL producing isolates were characterized phenotypically for ESBL production using the double disc synergy test.

**Results:**

In total, 383 specimens were assessed, among which 212 (55.4%) were related to bacteria producing ESBLs (ESBL+). Of those with ESBL + infections, 65.5% were sourced from catheters (as hospital-associated UTIs), and 35.5% were categorized as community-associated UTIs. In the group consisting of bacteria producing ESBLs, the high- est sensitivity was observed with Imipenem (72.2%), while the highest resistance was revealed with ceftriaxone (100%).

**Conclusion:**

We have shown that our community faces a high prevalence of bacteria producing ESBLs, mostly sourced from the catheterization of hospitalized patients. The highest bacterial sensitivity was observed with Imipenem, nitrofu- rantoin, and amikacin, while the highest resistance was found with ceftriaxone and cotrimoxazole, suggesting the inef- fectiveness of using the two latter antibiotics for eradicating these bacterial infections. On the other hand, a history of urinary catheterization and previous hospitalization were two main determinants of their presence, a finding which em- phasizes the importance of avoiding catheterization and hospitalization of patients with UTIs without proper indications.

## Introduction

The bacteria that infect the critically ill patients admitted into hospitals, especially into intensive care units, widely vary in terms of antibiotic resistance and genotypic pattern. Bacterial drug resistance is now a major concern leading to high morbidity, and even mortality, especially in third world countries (1). Due to the time-consuming nature of the laboratory diagnoses of these microbial agents, delays in diagnosis may lead to delays in proper antimicrobial therapy. There is also a growing concern regarding the lack of new antibiotics, especially for multidrug-resistant bacteria that produce ESBLs (2, 3). The *beta*-lactamases pro- duced by the Enterobacteriaceae family of gram-negative organisms, in particular *Klebsiella pneumonia *and *Escherichia coli *are hydrolytic enzymes that confer bacterial resistance to β-lactam antibiotics, such as the penicillin and cephalosporin families that are common antimicrobial drugs found all around the world (4, 5). The bacteria producing ESBLs have become a major cause of nosocomial infection, particularly in the ICU, with the majority of ESBL producers being isolated from critical care patients (6). Some risk factors have been identified that render patients prone to community-associated ESBL infections, including old age, being female, diabetes mellitus, previous antibiotic usage, recurrent urinary tract infections, and prior instru- mentation to urinary tract (7,8). Although a downward trend of infections due to ESBL-producing bacteria has been recently reported in developed countries, this trend has remained constant, or even increased in some developing countries (9). Unfortunately, the incidence of bacteria producing ESBLs remains uncertain. Hence, the present study aimed to assess the prevalence of bacteria producing ESBLs, their antimicrobial resistance pattern, and its main determinants among hospitalized patients in a great referral hospital in Iran between 2016 and 2017.

## Materials and Methods

The study targeted three endpoints included to determine the overall prevalence of ESBL (+) bacteria, to assess the antimicrobial resistance patterns of these species, and to determine the main predictors of the presence of these bacteria. In this regard, a biphasic study was planned as a cross-sectional study to achieve two former endpoints and a case-control study with the aim of comparing baseline variables between the two groups, with and without bacteria producing ESBLs. The study population included 383 consecutive patients, with a definitive diagnosis of urinary tract infections (UTIs), who were referred to Imam Khomeini hospital in Tehran within a period of five months. All eligible subjects for the study had a positive culture for gram negative microorganisms in urine specimens. Hospitalized patients over 18 years of age were categorized into two groups according to the criteria of the CDC guidelines as having 1) nosocomial UTIs: infections related to catheter insertion within hospitalization that was defined as a) having urine catheters for more than 2 days; b) having at least one of the following symptoms: fever (higher than 38ºC), suprapubic tenderness, tenderness in flank or abdomen, urinary urgency, frequency, or emergency; c) urine culture with a colony count higher than 105 colonies. 2) community-acquired UTIs: hospitalization due to UTIs with manifesting symptoms upon admission: including fever, urinary frequency, dysuria, urinary urgency, flank or suprapubic tenderness, and laboratory findings of UTIs including urine culture a colony count exceeding 105 colonies. All urinary specimens were collected mid-stream using the clean catch method via Foley catheter and were cultured in Mueller Hinton agar medium at 35ºC. Standard methods for isolation and identification of these bacteria were used. ESBL-producing isolates were characterized phenotypically for ESBL production by us- ing the Double Disc Synergy Test (DDST) as recommended by the Clinical Laboratory Standards Institute (CLSI) (10). The test was done by using cefotaxime (30μg) and ceftazidime (30μg), both alone and in com- bination with clavulanic acid. A >5 mm increase in zone diameter for either antimicrobial agents tested in combination with clavulanic acid, versus its zone when tested alone, was taken as positive result for ESBLproduction. To assess the factors that might be associated with the susceptibility to the infection by ESBL + bacteria, the baseline characteristics and clinical information of the patients, including demographics, medi- cal history, medications, and laboratory parameters, were collected by reviewing hospital records.

Results were presented as a mean ± standard deviation (SD) for quantitative variables and were summarized by absolute frequencies and percentages for categorical variables. The normality of data was analyzed using the Kolmogorov-Smirnoff test. Categorical variables were compared using the chi-squared test or Fisher’s exact test when more than 20% of cells with an expected count of less than 5 were observed. Quantitative variables were also compared with the t-test or the Mann-Whitney U test. The main determinants of the ESBL (+) condition were assessed using multivariable logistic regression modeling. For the statistical analysis, the statistical software SPSS version 16.0 for windows (SPSS Inc., Chicago, IL) was used. P values of 0.05 or less were considered statistically significant.

## Results

We assessed 383 specimens with definitive diagnoses of UTI, out of which 212 (55.4%) cases were ESBL (+).

The male to female ratio in the ESBL (+) group, compared to the ESBL (-) group, was significantly higher (*P*=0.002) (Table 1). There was no difference in mean age between these two groups (50.80 ± 22.24 years versus 47.79 ± 25.64 years, *P*=0.220) (table 1).

With regards to past medical history, there were more patients with diabetes in the ESBL(+) group com- pared to the ESBL(-) group (10.9% versus2.9%, *P*=0.003), the same as their history of cancer and chemo- therapy (20.8% versus 11.1%, *P*=0.011), renal failure and dialysis (14.2% versus 5.8%, *P*=0.041) , ICU admission (22.1% versus 9.3%, *P*= 0.001), long hospital admission of over seven days (14.1% versus 5.9%, *P*=0.008) and antibiotic consumption in the past 3 months (16% versus 2.9% , *P *<0.001) . A history of UTI in the past three months was also more in the group with bacteria producing ESBLs (*P*=0.012).

However, there was no between-group difference in the history of ischemic heart diseases, CVA and previ- ous surgeries in the urinary tract in the past 1 year.

The Foley catheter was significantly more used in the ESBL (+) group (61.8% versus 23.4%, *P*= 0.001). On the other hand, 65.5% of cases of the ESBL (+) group were sourced from catheters (as hospital-associated UTIs), and 35.5% were categorized as community-associated UTIs.

No difference was found in the prevalence of some species, such as *E. coli*, between two groups (64.6% versus 66.1%), while some other species. such as *Klebsiella*, were more prevalent in the ESBL (+) group (30.2% versus 21.6%).

**Figure1:**
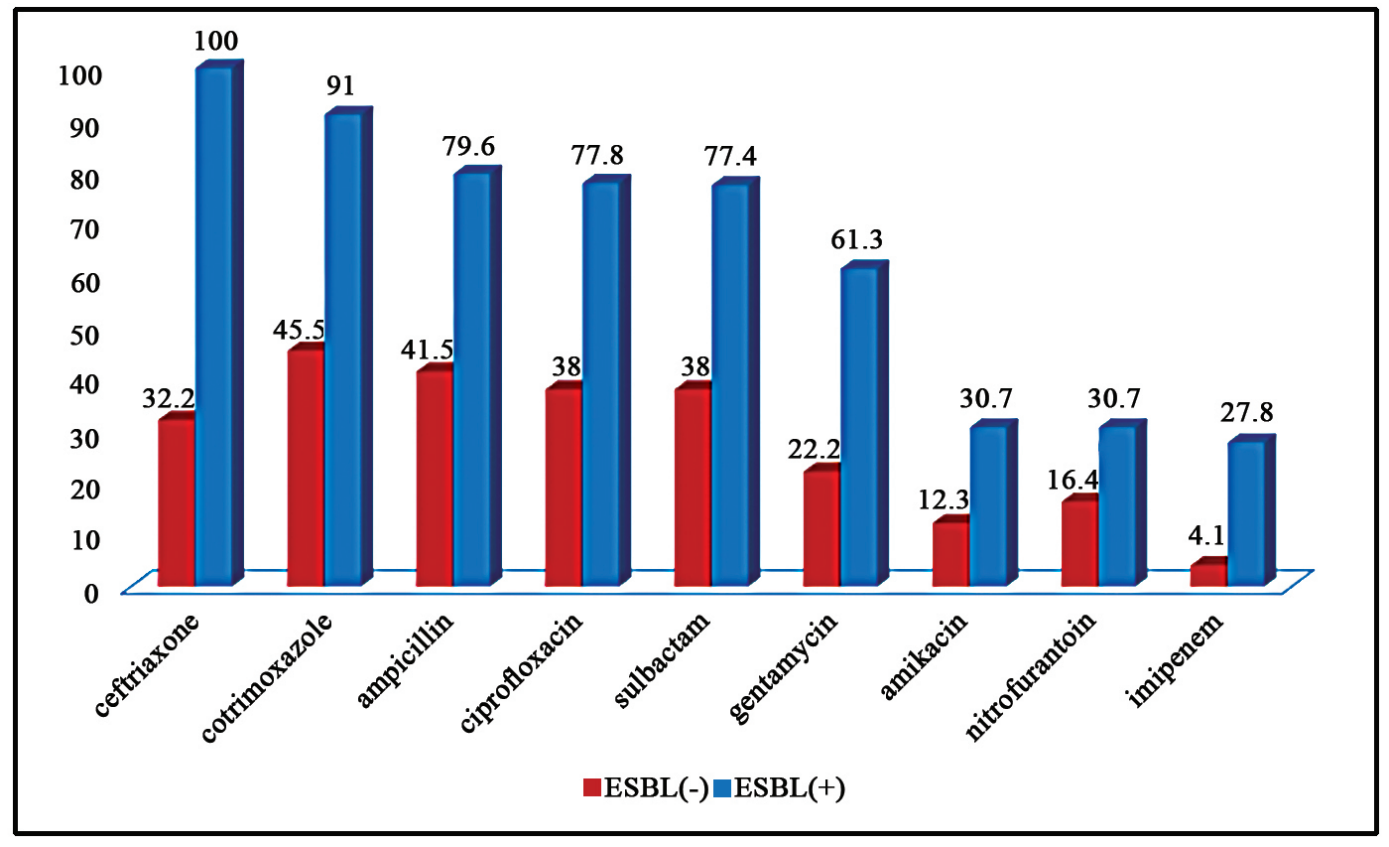
The resistance with different antibiotics in ESBLs (+) and ESBL (-) groups

In the group consisting of bacteria producing ESBLs, the highest sensitivity was observed with Imipenem (72.2%), followed by nitrofurantoin (69.3%), amikacin (69.3%), and gentamycin (38.7%) (Figure 1), while the highest resistance was revealed to be with ceftriaxone (100%) and cotrimoxazole (91.0%). As shown in Figure 1, the resistance to all types of antibiotics was considerably higher in the ESBL (+) group compared with the other group. Using the multivariate logistic regression model, and with the presence of potential confounders, the history of using Foley catheter (OR=3.761, *P*<0.001) and history of hospitalization (OR=5.590, *P*< 0.001) were the main predictors of the presence of ESBL-producing bacteria.

**Table 1:** Baseline characteristics between the groups with and without bacteria producing ESBLs

**Item**	**ESBL (+) group**	**ESBL (-) group**	***P*** **-value**
Male	40.1%	25.1%	0.002
Mean age, year	50.80 ± 22.24	47. 79 ± 25.64	0.220
Bacterial species			
E. Coli	64.6%	66.1%	0.879
Kelebsiella	30.2%	21.6%	0.001
Citrobacter	4.2%	2.9%	0.166
Enterobacter	0.0%	5.3%	0.098
Morganella	0.0%	0.6%	0.759
Proteus	0.0%	2.9%	0.456
Other enterobacteriace	0.9%	0.6%	0.579
Foley catheter used	61.8%	23.4%	0.008
Renal failure and dialysis	14.2%	0.6%	0.041
Ischemic heart disease	4.2%	2.3%	0.307
Cerebrovascular disease	3.3%	2.3%	0.576
cancers +chemotherapy	20.8%	11.1%	0.011
Diabetes mellitus	10.9%	2.9%	0.003
Surgery of urinary tract in past one Year ago	3.8%	2.3%	0.424
Admission more than 7 days	14.1%	5.9%	0.008
History of hospitalization in past 3 mo	47.2%	11.1%	< 0.001
History of antibiotic consumption in past 3 mo	16%	2.9%	< 0.001
History of UTI in past 3 mo	13.7%	5.9%	0.012
History of ICU admission	22.1%	9.3%	0.001

## Discussion

The proliferation of ESBL-producing bacteria is an important alarm for all countries around the world, due to their high resistance to various antimicrobial agents. In this regard, different factors such as underlying chronic disorders, a history of hospitalization, malignancies, urinary tract disorders, or urinary catheterization, as well as some mutations in bacterial DNAs, may increase the prevalence of these bacteria, not to mention their resis- tance to different antibiotics. Thus, the use of proper antibiotics, along with avoiding the overuse or misuse of antibiotics with a high resistance pattern, should be considered to minimize the development of these infections.

In the present study, the overall prevalence of ESBL-producing bacteria was shown to be 55.4% in our patient population, two-thirds of which was related to urinary catheterization in hospitalized patients, while the rest were community-acquired infections. Secondly, the highest prevalence rate ESBL-producing bacteria was re- lated to E.coli at 64.6%, followed by Klebsiella at 30.2%, which seems to be inconsistent with some previous reports. In a study by Sharma et. al. (11) in 2013 on Indian patients, 52.49% were found to be ESBL-producing. Also, among various isolates, the highest ESBL production was observed in Klebsiella pneumoniae (67.04%), followed by Escherichia coli (56.92%). In another study conducted in Tanzania, the overall prevalence of ESBLs in all Gram-negative bacteria was 29%. The ESBL prevalence was 64% in the Klebsiella pneumoniae species and 24% in E. coli (12).

Frightening figures were also obtained in a study in Mali, where 63% of adults and 100% of children were found to carry ESBL-producing Enterobacteriaceae (13). Moreover, in Madagascar, Herindrainy et al. (14) in 2011 observed that 10% of non-hospitalized patients carried ESBLs. Fatemeh et al. (15) also found that 26.5% of E. coli and 43% of Klebsiella pneumoniae were ESBL-positive in their study, which was conducted at the Imam Reza Hospital of Mashhad.

It was also shown that in the group consisting of ESBL-producing bacteria, the highest sensitivity was observed with Imipenem (72.2%), followed by nitrofurantoin (69.3%), amikacin (69.3%) and gentamycin (38.7%), while the highest resistance was observed with cotrimoxazole (91.0%) and ceftriaxone (100%).

In other words, sensitivity with almost all antibiotics was considerably low, even with beta-lactams and mac- rolides. In this regard, the resistance to cephalosporin and co-trimoxazole was considerably high, revealing the ineffectiveness of these two types of antibiotics in eliminating ESBL-producing bacteria from our hospitalized patients.

In a Sharma et al. survey (15), the sensitivity with Imipenem was found to be at 100%, and the highest sen- sitivity was shown to be with beta-lactams and aminoglycosides. Another investigation was conducted at a ter- tiary hospital in Nigeria by Ruth et al (16), in which the ESBL isolates showed high resistance to tetracycline, gentamicin, pefloxacin, ceftriaxone, cefuroxime, ciprofloxacin and Augmentin. In a study by Majda et al (17), maximum resistance was recorded in *E. coli *as cefotaxime (98.9%), Ceftazidime (96.7%) and Cefuroxime (93.4%) while minimum resistance was seen with Imipenem (0.8%), fosfomycin (1.2%) and nitrofurantoin as well piperacillin/tazobactam (2.2%) each.

The ESBL-producing *Klebsiella *showed maximum resistance to ceftazidime (100%), cefotaxime (89%), and Cefuroxime (84%) while minimum resistance was seen with Imipenem (4%), Nitrofurantoin and Piperacillin/ Tazobactam (8%).

In another study, Shakti et al (18) showed a progressive increase in drug resistance against each antibiotic, with the maximum resistance values recorded against gentamycin: 92% and 79%, oxacillin: 94% and 69%, ceftriax- one: 85% and 58% , and Norfloxacin 97%, and 69% , both in nosocomial and community isolates respectively.

Our study identified these conditions as risk factors for developing ESBL(+) infections: Foley catheterization, renal failure and dialysis, cancer and chemotherapy, ICU admission, DM, long admission exceeding 7 days, a history of UTI in the past 3 months, hospitalization in the past 3 months, and a history of antibiotic consumption.

In total, our community faces the high prevalence of ESBL-producing bacteria, most of which originate from catheterization among hospitalized patients. In addition, the highest bacterial sensitivity was related to Imi- penem, nitrofurantoin, and amikacin, while the highest resistance was found to be with ceftriaxone and co- trimoxazole. In the end, according to the potential effect of Foley catheterization, and prolonged or repeated hospitalization, on the prevalence rate of ESBL-producing bacterias, it is strongly encouraged to pay attention to the catheterization and hospitalization of patients with UTIs to minimize the number of these bacteria in the community.
